# Age-of-onset of hypertension vs. a single measurement of systolic blood pressure in a combined linkage and segregation analysis

**DOI:** 10.1186/1471-2156-4-S1-S80

**Published:** 2003-12-31

**Authors:** E Warwick Daw, Xiaoming Liu, Chih-Chieh Wu

**Affiliations:** 1 Department of Epidemiology, University of Texas MD Anderson Cancer Center, 1515 Holcombe Boulevard., Houston, Texas USA; 2 Program in Human and Molecular Genetics, University of Texas Graduate School of Biomedical Sciences, Houston, Texas USA

## Abstract

**Background:**

Often, multiple measures of a trait are available in a genetic linkage analysis. We compare Monte Carlo Markov chain analysis of two very different measures of hypertension in the simulated Genetic Analysis Workshop 13 data to examine how choice of measure affects the results. The measures selected were age-of-onset of hypertension and systolic blood pressure at first visit.

**Results:**

In combined segregation and linkage analysis of the complete pedigrees using the first replicate of the simulated data with missing values, we found that the age-of-onset analysis was better at identifying "slope" genes, while the systolic blood pressure analysis was better at identifying "baseline" genes.

**Conclusion:**

Analysis of different trait measures may identify different trait-related genes. When linkage analysis is conducted on multiple trait measures, a linkage signal found for only one measure can represent a true trait locus.

## Background

In studies such as the Framingham Heart Study (e.g. [1]) or the Collaborative Study on the Genetics of Alcoholism (COGA) [2], a number of different values related to a disease are measured, and the first step in the analysis may be choosing one measure. The simulated data in Genetic Analysis Workshop 13 (GAW13), for example, offered many measures related to hypertension. Exactly which measure one uses may depend on the analysis method, heritability estimates, and clinical knowledge of the mechanisms behind the disease. Sometimes more than one measure is analyzed and different results are obtained for the different measures, as was see in the GAW11 analyses of the COGA data [3].

Here, we compared two different simulated measures of hypertension in analyses with Monte Carlo Markov chain (MCMC) oligogenic combined segregation and linkage analysis, as implemented in the program Loki [4]. These methods use linkage data on extended pedigrees and estimate the number, location, and effects of loci that contribute to a quantitative trait. We chose two measures of a type that might be collected in a retrospective study: age-of-onset of hypertension (AOH) and systolic blood pressure at the first visit (SBP). We made these choices with knowledge of the generating model. We wanted to determine whether these different measures would localize different trait loci.

## Methods

### Trait selection

From the first replicate of the simulated GAW13 data, we selected two traits that were related, but had slightly different genetic characteristics. Both simulated traits we selected, AOH and SBP at the first visit, are related to hypertension. However, since the generating model divided the trait loci into those that affected "baseline" value and those that affected "slope," we expected that SBP would localize "baseline" genes, while AOH would localize "slope" genes. For SBP we used the SBP value from the first examination in both cohorts. We used the simulated data with missing values. The data set did not contain age-of-onset data as a separate value, but it did contain an indicator of hypertension diagnosis at each visit. We used the age at the earliest visit with a hypertension diagnosis as AOH. Since "baseline" was age 20 at the generating model and the first visit occurred at different ages for different subjects, our SBP measure was not a pure measure of the "baseline." Similarly, since AOH was determined by the crossing of a threshold, AOH is not a pure measure of "slope." Such "impure" measures may, however, better reflect the measures found in studies of real data.

### MCMC segregation and linkage analysis

To estimate the number, effects, and location of loci contributing to AOH and SBP, we applied the MCMC segregation and linkage analysis methods described in Heath [4] and Daw et al. [5]. These methods also estimate covariate effects, and the trait model is given by



where μ is the "reference" trait value, *X *is the incidence matrix for covariate effects, β is the vector of covariate effects, *Q*_*i *_is the incidence matrix for the effects of QTL *i*, α_*i *_is the vector of effects for QTL *i*, *e *is the normally distributed residual effect, and *k *is the number of QTL currently estimated (*k *≥ 0). The MCMC process samples μ, β, α_*i*_, *i*, and *e *as well as parameters such as unobserved marker genotypes. All these parameters are sampled from the space of model values consistent with the data observed. Values are sampled proportional to their posterior probability. After the number of sampling iterations is sufficiently large, the sampled values provide an estimate of the posterior probability distribution over the space of possible parameter configurations. We chose a set of covariates that had an effect in the generating model. For comparability, nearly the same covariates were used for both traits. For SBP, the covariates included cigarettes smoked per day (CPD), sex, and age, as reported at the first visit. For AOH, the effects of CPD and sex were estimated as covariates. AOH covariates are a subset of those for SBP, with only age not used in the case of AOH. The censored trait model described in [5] was used for AOH. This censoring model is essentially a genetic survival analysis with cumulative normal survival curves. Thus, the age information still contributes in both of our analyses.

We conducted a complete genome scan for both traits. We first carried out analyses on both models on all 22 chromosomes using 50,000 iterations, while saving every fifth iteration. On chromosomes with evidence for linkage, we followed up with longer runs. Additionally, for each trait we conducted a longer multi-chromosome analysis including all chromosomes with an L score > 5. These longer runs were 200,000 to 500,000 iterations in length. All analyses were conducted with the sex-averaged Haldane map provided with the simulated data.

### Bayesian "L-score"

To evaluate evidence for linkage, we considered Bayes factors estimated over 1-cM wide bins along the chromosomes. A Bayes factor is simply the posterior probability divided by the prior probability. In the absence of any data, a Bayesian analysis should have posterior probability equal to the prior probability. Thus, a Bayes factor of 1 indicates that the data contains no information for or against linkage. A Bayes factor < 1 indicates evidence against linkage, while a Bayes factor > 1 indicates evidence for linkage. We refer to these Bayes factors for linkage calculated in 1-cM intervals as "L-scores." We used the Haldane map provided with the simulated data.

## Results

In both analyses, there were three regions with L-scores that stood out with values > 50, while the next largest scores were < 25. All three regions that were identified in this way contained simulated trait loci (see Table 1). As expected, the largest L-score in the AOH analysis was at the largest-effect "slope" gene, Gs10. The peak score occurred in a bin adjacent to the one actually containing the locus, so the localization of the gene was within 2 cM of the true location. Also as expected, the largest L-score in the SBP analysis was at the largest effect SBP baseline gene, Gb34, although the peak was some 8 cM away from the true gene location. The second largest "baseline" gene, Gb35, was also identified in the SBP analysis. Of particular interest, the SBP analysis also identified Gs10, although the L-score was somewhat smaller than the AOH analysis, and the peak value was further from the true gene location than in the AOH analysis. The AOH analysis found weak evidence for linkage at Gb34 and no evidence for linkage at Gb35.

Exactly where to draw a threshold for follow-up is not clear. In addition to the three locations with L-scores > 50, there were several regions with L-scores > 10 and < 50. For completeness, we list all regions with L-scores > 10. For AOH, these L-scores were: ~21 at ~80 cM on chromosome 5, ~22 at ~95 cM on chromosome 7, ~15 at 45 cM on chromosome 14, and ~11 at ~50 cM on chromosome 19. For SBP, these L-scores were: ~14 at ~220 cM on chromosome 3 and ~11 at ~95 cM on chromosome 16. Since no trait loci were placed on even-numbered chromosomes, the two signals on even numbered chromosomes represent weak false-positives. It is more difficult to say anything conclusive about the signals on the odd-numbered chromosomes because of the complexity of the generating model: all of the odd numbered chromosomes do, in fact, contain loci that contribute to the simulated hypertension trait. Since more signals in this range of 10 to ~20 are on odd numbered chromosomes, it seems likely that at least some of these are true weak positive signals. The largest effect height gene, Gb1, is at ~80 cM on chromosome 5, while a total of nine different genes are on chromosome 7, making these signals for AOH likely true weak positives and both of these are over 20. Any threshold value may depend on the investigators tolerance of false-positives, but while the three strong signals (Table 1) were all > 50, the next best signal was < 25, and the largest score on an even-numbered chromosome was ~15.

## Conclusions

We find that analysis of AOH is better at localizing slope genes, while analysis of SBP is better at identifying baseline genes. We noted with interest at GAW 13 that some other groups reported better localization of slope genes with methods that examine value at one time-point rather than "slope." We believe we were able to identify Gs10 in our SBP analysis because most subjects were over the baseline age of 20 at the first visit and the slopes were generally positive, causing the variation in SBP to increase with age. Some alternate generating models were suggested at the GAW 13 meeting under which we would expect our SBP analysis to fail to localize slope loci. Exactly what cut-off should be used for declaring significance with L-scores remains an open question. Our results suggest that analysis of different trait measures can identify different trait loci. Thus, if one has multiple measures, conducts linkage analyses on all of them, and only focuses on those linkage signals replicated in analyses of several measures, one may miss some trait loci.

## Figures and Tables

**Table 1:** Locations of largest L-scores in single chromosome analysis of AOH and SBP^A^

		AOH^B^		SBP	
		
QTL	Location	L-score	Peak	L-score	Peak
Gs10	53.59 cM – chr. 21	94.5	52 cM	76.7	49 cM
Gb34	176.08 cM – chr. 5	4.8	180 cM	84.4	168 cM
Gb35	85.16 cM – chr. 13	0.3	93 cM	59.4	94 cM

^A^Values and peak locations for largest L-scores. ^B^L-score values for AOH analysis at Gb34 and Gb35 included for comparison.
